# Mathematical modeling of left ventricular dimensional changes in mice during aging

**DOI:** 10.1186/1752-0509-6-S3-S10

**Published:** 2012-12-17

**Authors:** Tianyi Yang, Ying Ann Chiao, Yunji Wang, Andrew Voorhees, Hai-Chao Han, Merry L Lindsey, Yu-Fang Jin

**Affiliations:** 1 San Antonio Cardiovascular Proteomics Center, The University of Texas Health Science Center at San Antonio, USA; 2 Department of Electrical and Computer Engineering, The University of Texas at San Antonio, USA; 3 Barshop Institute of Longevity and Aging Studies; Division of Geriatrics, Gerontology and Palliative Medicine, Department of Medicine, The University of Texas Health Science Center at San Antonio, USA; 4 Department of Biochemistry, The University of Texas Health Science Center at San Antonio, USA; 5 Department of Mechanical Engineering, The University of Texas at San Antonio, USA

## Abstract

Cardiac aging is characterized by diastolic dysfunction of the left ventricle (LV), which is due in part to increased LV wall stiffness. In the diastolic phase, myocytes are relaxed and extracellular matrix (ECM) is a critical determinant to the changes of LV wall stiffness. To evaluate the effects of ECM composition on cardiac aging, we developed a mathematical model to predict LV dimension and wall stiffness changes in aging mice by integrating mechanical laws and our experimental results. We measured LV dimension, wall thickness, LV mass, and collagen content for wild type (WT) C57/BL6J mice of ages ranging from 7.3 months to those of 34.0 months. The model was established using the thick wall theory and stretch-induced tissue growth to an isotropic and homogeneous elastic composite with mixed constituents. The initial conditions of the simulation were set based on the data from the young mice. Matlab simulations of this mathematical model demonstrated that the model captured the major features of LV remodeling with age and closely approximated experimental results. Specifically, the temporal progression of the LV interior and exterior dimensions demonstrated the same trend and order-of-magnitude change as our experimental results. In conclusion, we present here a validated mathematical model of cardiac aging that applies the thick-wall theory and stretch-induced tissue growth to LV remodeling with age.

## Introduction

Over 70% of 50 million Americans over 60 years of age have cardiovascular disease (CVD) [1,2]. CVD prevalence increases with age, and outcomes in older patients with acute coronary syndromes are poor [1,3]. It is important to note, however, that aging itself, even in the absence of CVD, alters LV structure and function and impairs the ability of the LV to respond to stress and injury. Thus, understanding the mechanisms of cardiac aging has significant clinical relevance.

While most studies focus on the myocyte contribution to cardiac systolic function, indices for systolic function, such as ejection fraction, systolic velocities, and systolic isovolumic acceleration rate, have been shown to have little relation with age in both clinical and animal studies [4,5]. In contrast, echocardiographic indices of diastolic function including early (E) and late (Atrial-A) diastolic peak filling velocities and the E and A velocity ratio (E/A ratio), mitral deceleration time (the time from the peak to the end of the Doppler E-wave), isovolumic relaxation time (the time between the closing artifact of the aortic valve), and the earliest detection of trans-mitral blood flow have been demonstrated to decline with age in clinical studies [6-8]. With physiological aging, the LV undergoes monotonic structural changes that include increased wall thickness, chamber diameter, and mass [6-8].

We have shown diastolic dysfunction at the organ level in mice during cardiac aging [9], and diastolic dysfunction is caused by increased myocardial stiffness at the tissue level [10]. The myocardium is composed of myocytes (muscle) surrounded by the ECM environment. Accordingly, myocardial stiffness is determined by the volume ratio and the combined mechanical property of the myocytes and ECM. In the diastolic phase, myocytes are relaxed and ECM is a critical determinant to the change of LV wall stiffness. About 90% of cardiac ECM composition in the young LV is collagen I and III. We have shown that collagen content in senescent mouse hearts doubles compared to young hearts [9,11]. Since collagen has a magnitude increase in stiffness over myocytes, the age-related increase of collagen content in the LV shifts myocardial mechanical properties from a myocyte-based stiffness to one influenced by collagen-based stiffness [12]. Therefore, the goal of this study was to evaluate the effects of ECM composition on LV remodeling with aging using a mathematical model developed by integrating cardiac mechanics and our experimental results in mice.

Different LV wall stress models, such as the Laplace law based thin-wall models, thick-wall shell models, and finite element models, have been established to describe LV mechanics and compute stress [13,14]. Most current thin-wall and thick-wall models of LV remodeling were established assuming idealistic spherical, spheroidal, or ellipsoidal geometries. While finite element models allow some flexibility in the LV geometry, they require high computational power. Currently, these models focus on the stress calculation taken from a particular snapshot of the cardiac cycle or for the entire cycle. However, the interplay between LV stress and strain and between remodeling and geometric evolution on the life-time scale has not been established.

Phenomenological models on geometric remodeling with aging have been developed to apply on arteries under hypertensive conditions [15,16]. The arterial wall is considered as a thick-wall vessel, and the remodeling equation on either inner or outer artery surface has been postulated as a function of strain and stress at the corresponding location. While these models do not consider the intrinsic relationships between phenomenological assumptions, they provide a possible methodology to model the temporal progression of tissue remodeling with aging.

There are very few computational models available to study LV geometric adaptation with aging. Recently, we established a computational model of LV aging that incorporates Laplace law based stress model [11]. This model captures the overall trend of LV radius change with age. However, this thin-wall model assumes a constant thickness and no stress variation across the wall, LV thickening with age was not addressed. Here, we improve this model by adopting a more sophisticated thick-wall theory and stretch-induced tissue growth theory for the model used in this study. The novelty of this model lies in the integration of both computational and experimental approaches. The wall radii remodeling model established in this study was subject to the temporal function of the total mass which was measured in our experiments. Additionally, the predictions of LV geometry and end diastolic pressure-volume relation from the mathematical model were compared with our experimental measurements to validate predictions of the mathematical model.

## Methods

With physiological aging, the LV undergoes tonic structural and functional changes including increased mean LV wall thickness, chamber diameter, mass, concentric remodeling, and decline in LV diastolic function in humans [6-8]. We assumed mice age-related remodeling follow similar rules. Our model was established based on the following 2 assumptions.

1) The LV was modeled as a concentric spherical shell with interior and exterior radii $R_{i}$ and $R_{o}$, respectively, at the no-load state. Due to the transmural pressure difference P on the inner and outer surfaces, the deformed inner and outer radii were $r_{i}$ and $r_{o}$, respectively. The ventricular wall was modeled as an isotropic and linear elastic material of two constituents, cardiac myocytes and ECM which is represented by collagen.

(2) Internal and external radii, ie., *R_i _*and *R_o_*, as well as wall thickness *R_o _- R_i_*, all have non-decreasing trend with age.

### Thick-wall LV model

Based on assumption 1), deformation of the LV wall obeys Hook's law, which can be expressed in tensor form: $\in_{ij}=\frac{1+\nu }{E}\sigma_{ij}-\frac{\nu }{E}\delta_{ij}\sigma_{kk}$, where *∈ *is strain tensor, *σ *is stress tensor, *v *is Poisson's ratio, *E *is material's Young's modulus, *δ_ij _*is Kronecker delta, *i*, *j*, *k *∈ {*R*, *θ*, *φ*} are the spatial indices for radial, meridional, and circumferential directions. The strain in the radial direction is:

(1) $$\in_{RR}=\frac{\partial r}{\partial R}-1=\frac{1}{E}\left[\sigma_{RR}-\nu \left(\sigma_{\theta \theta }+\sigma_{\phi \phi }\right)\right].$$

The stresses for elastic, isotropic, homogeneous, and spherical thick-wall contains the following analytical form[17]:

(2) $$\sigma_{RR}\left(R\right)=\frac{PR_{i}^{3}}{R_{o}^{3}-R_{i}^{3}}\left(1-\frac{R_{o}^{3}}{R^{3}}\right),$$

(3) $$\sigma_{\theta \theta }\left(R\right)=\sigma_{\phi \phi }\left(R\right)=\frac{PR_{i}^{3}}{R_{o}^{3}-R_{i}^{3}}\left(1+\frac{1}{2}\frac{R_{o}^{3}}{R^{3}}\right),$$

in which, P denotes pressure difference applied to the inner and exterior walls, and *R *∈ [*R_i_*, *R_o_*]. Applying equations (2) and (3) to equation (1), we had

(4) $$\frac{\partial r}{\partial R}=1+\frac{P}{E}\frac{1}{{\left(R_{o}/R_{i}\right)}^{3}-1}\left[\left(1-2\nu \right)-\left(1+\nu \right){\left(R_{o}/R\right)}^{3}\right].$$

Integrate both sides of Eq. (4) from *R_i _*to *R_o_*, we obtained

(5) $$r_{o}-r_{i}=\left\{1-\frac{P}{E}\frac{1}{{\left(R_{o}/R_{i}\right)}^{3}-1}\left[\left(1+\nu \right)\frac{{\left(R_{o}/R_{i}\right)}^{2}+R_{o}/R_{i}}{2}-\left(1-2\nu \right)\right]\right\}\left(R_{o}-R_{i}\right).$$

It's concluded that Poisson's ratios of all stable isotropic materials falls in the region (-1, 0.5) and only auxetic materials with honeycomb structures and networks have been found to have negative values [18]. Apparently, heart tissue is not auxetic. Therefore, for LV, its Poisson's ratios falls in the region 0 <*v *< 0.5. As a consequence, it is apparent that the loaded wall thickness, i.e. *r_o _- r_i _*is smaller than the zero-load thickness, i.e. *R_o _- R_i_*. The LV transmural pressure P has been shown to alter with aging. We adopted the temporal profile of pressure difference reported in the literature [11,19].

The Young's modulus *E *increases with aging due to changes in the LV constituents. We previously reported that the fractions of the two constituents, i.e. myocytes and collagen changes with aging.[9,11] In this study, we adopted a simplified version of the linear mixture theory of composite material, Young's modulus obeys the following equation [20,21]:

(6) $$E\left(t\right)=E_{c}v_{c}\left(t\right)+E_{m}\left[1-v_{c}\left(t\right)\right],$$

in which *E_c _*and *E_m _*are two constants representing Young's moduli of collagen and muscle, respectively. Function *v_c_*(*t*) is the volume fraction of collagen, which can be expressed as:

(7) $$\upsilon_{c}\left(t\right)=\frac{V_{c}\left(t\right)}{V_{\text{c}}\left(t\right)+V_{m}\left(t\right)}=\frac{M_{c}\left(t\right)/\rho_{\text{c}}}{M_{\text{c}}\left(t\right)/\rho_{c}+M_{m}\left(t\right)/\rho_{m}}.$$

In equation (7), *V_c_*(*t*)*/V_m_*(*t*) is the volume occupied by collagen/muscle at time *t*, and *M_c_*(*t*)/*M_m_*(*t*) is the mass of collagen/muscle at time *t*. Parameters *ρ_c _*and *ρ_m _*are mass densities of collagen and myocytes. The density of collagen is an intrinsic property of the material and therefore does not change with time unless the collagen molecular structure undergoes significant changes. The density of collagen is adopted as *ρ_c _*= 1.70 g/ml [22]. For muscle, due to the fact that approximately 80% of the mass is water as has been measured in experiments for multiple mammalian muscle (either cardiac or non-cardiac), the density is close to that of water, with variation contributed by tissue and tissue solids. We adopt myocyte density as *ρ_m _*= 1.06 g/ml [23,24].

Due to the fact that both collagen and muscle mass evolve with aging, the volume of the wall changed accordingly. The mass evolution enforces the following equations:

(8) $$M_{\text{c}}\left(t\right)=\rho_{c}\upsilon_{\text{c}}\left(t\right)\int_{R_{i}\left(t\right)}^{R_{o}\left(t\right)}4\pi R^{2}dR,$$

(9) $$M_{m}\left(t\right)=\rho_{m}\left[1-\upsilon_{\text{c}}\left(t\right)\right]\int_{R_{i}\left(t\right)}^{R_{o}\left(t\right)}4\pi R^{2}dR.$$

Take the derivative of equations (8) and (9) with respect to time, we obtained

(10) $${Ṁ}_{c}\left(t\right)/\rho_{c}=4\pi \upsilon_{c}\left(t\right)\left[R_{o}^{2}\left(t\right){Ṙ}_{o}\left(t\right)-R_{i}^{2}\left(t\right){Ṙ}_{i}\left(t\right)\right]+\frac{4\pi }{3}\left[R_{o}^{3}\left(t\right)-R_{i}^{3}\left(t\right)\right]{\dot{\upsilon }}_{c}\left(t\right),$$

(11) $${Ṁ}_{m}\left(t\right)/\rho_{m}=4\pi \left[1-\upsilon_{c}\left(t\right)\right]\left[R_{o}^{2}\left(t\right){Ṙ}_{o}\left(t\right)-R_{i}^{2}\left(t\right){Ṙ}_{i}\left(t\right)\right]-\frac{4\pi }{3}\left[R_{o}^{3}\left(t\right)-R_{i}^{3}\left(t\right)\right]{\dot{\upsilon }}_{c}\left(t\right).$$

Add up Eqs. (10-11) and divide both sides by 4*π*, the following equation about free radii was obtained:

(12) $$R_{o}^{2}\left(t\right){Ṙ}_{o}\left(t\right)-R_{i}^{2}\left(t\right){Ṙ}_{i}\left(t\right)=\frac{{Ṁ}_{c}\left(t\right)+\frac{\rho_{\text{c}}}{\rho_{m}}{Ṁ}_{m}\left(t\right)}{4\pi \rho_{c}}.$$

Wall incompressibility yields:

(13) $$r_{o}^{3}\left(t\right)-r_{i}^{3}\left(t\right)=R_{o}^{3}\left(t\right)-R_{i}^{3}\left(t\right).$$

Since we assumed that the material is isotropic and homogeneous in our model (assumption 3), the spatial remodeling can only occur in the radial direction. As a result, the spherical geometry is always maintained. Therefore, only radii, e.g., the free radii *R_i _*and *R_o _*alter with aging.

### Stretch-induced tissue growth model

The dynamic equation that governs the temporal behavior of the exterior radius in the free state was defined as $\alpha \left(t\right):=\frac{R_{o}\left(t\right)}{R_{o}\left(t_{0}\right)},$ which is the ratio of free exterior radius to its initial value. Further, since the deformed wall thickness is smaller than that in the free state, volume conservation between free state and deformed state of LV wall guaranties *r_o _*>*R_o _*and *r_i _> R_i_*. Additionally, the ratio between deformed and free exterior radii $\lambda \left(t\right):=\frac{r_{o}\left(t\right)}{R_{o}\left(t\right)}$ is always larger than 1 (λ(*t*) > 1) since *r_o _*>*R_o_*. We proposed that the rate of change of free exterior radius had a power law relation kernel with the ratio λ(*t*) and the curve followed an overall exponential decay,

(14) $$\dot{\alpha }\left(t\right)=f\left(t\right)=\tau_{R}^{-1}\left[\lambda^{D}\left(t\right)-1\right]\;exp\;\left[-\left(t-t_{0}\right)/\tau_{f}\right],$$

in which, *f *(*t*) is the function that characterized the rate of change for the outer radius, parameter *τ_R _*is the remodeling characteristic time for *R_o_*(*t*), variable $\lambda^{D}\left(t\right)-1=\frac{ro^{D}\left(t\right)-Ro^{D}\left(t\right)}{Ro^{D}\left(t\right)}$ is the ratio of the change between deformed and free radii raised to the power *D *on the exterior surface, parameter *τ_f _*is the decay constant for function *f *which guarantees a bounded outer dimension of the LV. The parameters *τ_R_*, *D *and *τ_f _*are constant parameters to be determined.

Equations (5-7) and (12-14), together with the definitions of *α*(*t*) and λ(*t*), formed a mathematical model to characterize the temporal progression of the LV geometry by applying thick-wall theory and stretch-induced tissue growth. Given the temporal functions *M_c_*(*t*), *M_m_*(*t*) and *P*(*t*) with aging, we solved the mathematical model numerically.

### Input functions and parameters

**Figure 1:**
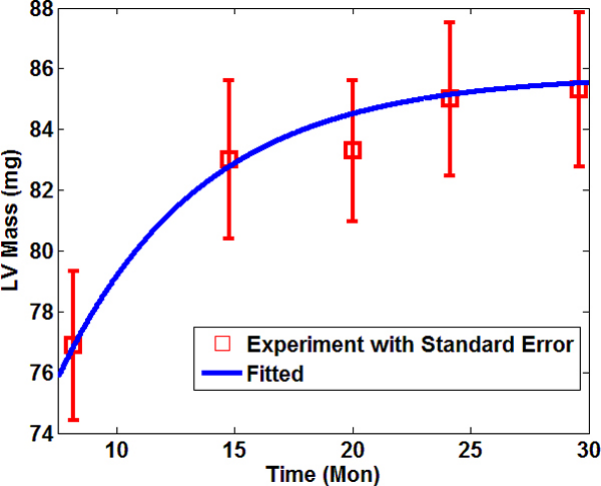
**Fitted temporal profile of the LV mass with age**. Red rectangles denoted the average values of LV mass at the ages of 8, 15, 20, 24, and 29.5 months collected over 140 wild type C57BL6J mice. Mass was measured by necropsy. The solid curve was the fitted temporal profile based on our experimental data.

**Figure 2:**
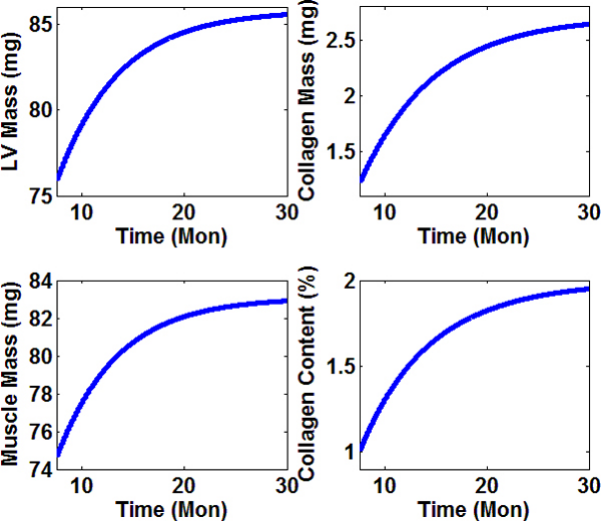
**Computational results for mass functions for LV, collagen, and muscle, as well as volume fraction for collagen**. These functions were used as inputs to our mathematical model. (A) The total LV mass fitted with data generated in autopsy experiments; (B) Collagen temporal profile fitted with known trend from experiments; (C) Temporal profile of muscle mass calculated as the difference between total and collagen mass; (D) Volume fraction of collagen, the values for young (6-9 months) and senescent (>25 months) were obtained from published results of mouse.

*M_c_*(*t*), *M_m_*(*t*), and *P*(*t*) are temporal functions established by fitting experimental data. We measured the LV mass of 148 wild type mice at different ages by necropsy. We adopted a smooth monotonic function that saturates with time to fit the experimental data for LV mass. The total LV mass function used in our simulation is *M*(*t*) = 76.88 + 8.92 {1 - *exp*[- (*t *- 8.18)/*τ_LV_*} (solid line in Figure 1), the mass unit is milligram, the unit of time *t *is months, and *τ_LV _*= 6.08 months. One of our recent studies showed that the collagen content (volume fraction) in wild type C57/BL6J mice is doubled in senescent mice (~1%) compared to young mice (~0.5%). Our previous studies also demonstrated the same trend of collagen content increase [11]. In our model, we chose 1% collagen content for young at 7.5 months, and approaching 2% for old mice after 30 months. By adopting the converted collagen content, LV mass function *M*(*t*) above, and corresponding densities (see Table 1), the temporal curve for collagen mass was $M_{c}\left(t\right)=1.21+1.50\left\{1,-,e,x,p,[,-\left(t-7.50\right)\text{/}\tau_{c}\right\}$, with $\tau_{c}=7.23$ months and other units are the same to relevant ones in $M\left(t\right)$. Since our model assumed that the LV consisted of two constituents (assumption 4), muscle mass were calculated by subtracting collagen mass from the total LV mass as $M_{m}\left(t\right)=M\left(t\right)-M_{c}\left(t\right)$. Figures 2 showed the temporal profiles of the mass of the total LV, collagen, and myocytes, as well as collagen volume fraction with age. Based on our computational prediction, total LV mass underwent a 12.8% increase from 7.5 to 30 months. The mass growth for collagen was about 121% in parallel with a twofold increase in volume fraction as shown in Figure 2D due to a higher density in relative to muscle. The myocytes mass grew about 11.1% in senescent mice compared to young group.

**Table 1:** Constant parameters and initial values adopted in numerical calculation and the source of the data.

Parameters (unit)	Values	References
Initial value *R_i_*(*t *= 7.5*mons*) (mm)	2.0	Our experiments on over 148 mice
Muscle density *ρ_m _*(gcm^-3^)	1.06	[23,24]
Collagen density *ρ_c _*(gcm^-3^)	1.70	[22]
Muscle elastic modulus *E_m _*(Pa)	5.0×10^4^	[28]
Collagen elastic modulus *E_c _*(Pa)	3.2×10^7^	[29,30]
Poisson' ratio *v*	0.47	[31]
Time step *h *(hrs)	3	

**Figure 3:**
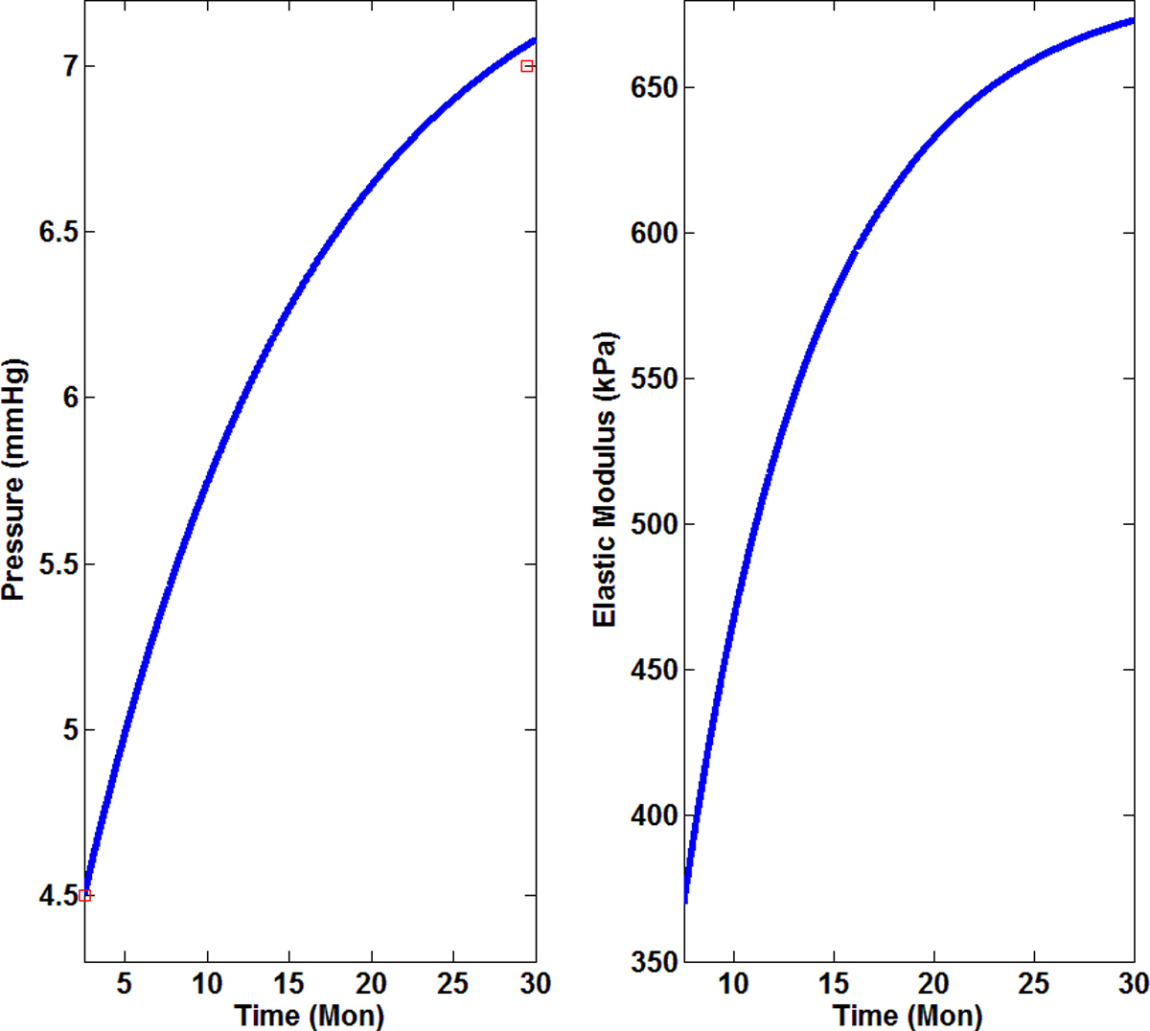
**Temporal profile of pressure difference of LV wall and predicted Young's modulus of the myocardium**. (A) Time-dependent pressure difference across the LV. The curved is derived by fitting experimental data; (B) Elastic modulus of LV, which was calculated as isotropic and homogeneous composite made up of muscle and collagen. The modulus was calculated by taking account of temporal profiles of muscle and collagen in LV.

Time-dependent pressure difference between the interior and exterior surfaces of the LV was another input function. We collected the pressure measurement in the literature and derived its temporal progression as $P\left(t\right)=4.5+3.0\left\{1-exp\left[-\left(t-2.5\right)\text{/}\tau_{p}\right]\right\}$ with unit mmHg (Figure 3A), where $\tau_{p}=14.0$ months [11,19]. The pressure ranged from 4.5 mmHg at 2.5 months to 7.1 mmHg at 30 months. We also showed the calculated Young's modulus of LV wall by assuming linear and homogeneously mixed composite made of muscle and collagen (Figure 3B). The modulus increased by 82% in senescent mice compared to young mice. Though there is only an increase from 1% to 2% increase of collagen volume ratio, it gives rise to significant enlargement in the modulus of the composite because the elastic modulus of collagen is much larger compared that of the muscle.

Further, from equations (2-3), the pressure difference P is the only external load that deforms the LV wall. With retreat of this pressure, or establishment of balance between inner and outer surfaces, any stress or strain disappears. Now if we compare the elastic modulus and the pressure difference, the highest pressure, i.e. 7.1 mmHg, is equivalent to 944 Pa, less than 2 orders of magnitude lower the magnitude of Young's modulus. Therefore, without detailed computation, we can see that there should exist none noteworthy deformation (< 1%) and the strained dimensions should be extremely close to the free ones. This was proved by detailed computation shown later.

### Discretization of equations

For convenience in the writing of equations, we defined $b\left(t\right)=\frac{{Ṁ}_{\text{c}}\left(t\right)+\frac{\rho_{\text{c}}}{\rho m}{Ṁ}_{m}\left(t\right)}{4\pi \rho_{\text{c}}}$, $c\left(t\right)=\left\{1-\frac{P\left(t\right)}{E\left(t\right)}\frac{1}{R_{i}^{o}{\left(t\right)}^{3}-1}\left[\left(1+\nu \right)\frac{R_{i}^{o}{\left(t\right)}^{2}+R_{i}^{o}\left(t\right)}{2}-\left(1-2\nu \right)\right]\right\}\left[R_{o}\left(t\right)-R_{i}\left(t\right)\right]$, in which $R_{i}^{o}\left(t\right)=R_{o}\left(t\right)/R_{i}\left(t\right)$, and $d\left(t\right)=R_{o}^{3}\left(t\right)-R_{i}^{3}\left(t\right)$. Now Equations (5), (12), and (13) can be rewritten as:

(15) $$R_{o}^{2}\left(t\right){Ṙ}_{o}\left(t\right)-R_{i}^{2}\left(t\right){Ṙ}_{i}\left(t\right)=b\left(t\right),$$

(16) $$r_{o}\left(t\right)-r_{i}\left(t\right)=c\left(t\right),$$

(17) $$r_{o}^{3}\left(t\right)-r_{i}^{3}\left(t\right)=d\left(t\right).$$

Given *R_i_*(*t*), *R_o_*(*t*), *P*(*t*) and, *E*(*t*) variables *c*(*t*) and *d*(*t*) could be determined and *r_i_*(*t*) and *r_o_*(*t*) can be solved as:

(18) $$r_{i}\left(t\right)=\frac{-c\left(t\right)+\sqrt{\left[4d\left(t\right)/c\left(t\right)-c^{2}\left(t\right)\right]/3}}{2},$$

(19) $$r_{o}\left(t\right)=\frac{c\left(t\right)+\sqrt{\left[4d\left(t\right)/c\left(t\right)-c^{2}\left(t\right)\right]/3}}{2}.$$

The radii $r_{i}\left(t\right)$ and $r_{o}\left(t\right)$ were real positive numbers subject to the condition *d*(*t*) >*c*^3^(*t*).

We studied the variation of free and deformed radii in the time domain [*t*_0_, *t*_0 _+ *T*] and evenly divided this region T by a total of N small constant intervals of *h*. Equivalently, the domain could be rewritten as [*t*_0_, *t*_0 _+ *Nh*]. The following is the procedure to calculate time-dependent radii.

1. Read initial values *R_i_*(*t*_0_), *R_o_*(*t*_0_), calculate *r_i_*(*t*_0_) and *r_o_*(*t*_0_) and $\lambda \left(t_{0}\right)=\frac{r_{o}\left(t_{0}\right)}{R_{o}\left(t_{0}\right)}$, note that *α*(*t*_0_) = 1. Iterate steps 2-5 until the final step *N*.

2. According to discretized equation (14) using Euler method, perform the following compute:

(20) $$\alpha \left(n+1\right)=\alpha \left(n\right)+\frac{\lambda^{D}\left(n\right)-1}{\tau_{R}}exp\left(-nh/\tau_{f}\right)h,$$

(21) $$R_{o}\left(n+1\right)=\alpha \left(n+1\right)R_{o}\left(t_{0}\right).$$

3. Utilize discretized version of Eq. (15) taking account of Eq. (14) to calculate the *R_i _*at the next time step.

(22) $$R_{i}\left(n+1\right)=R_{i}\left(n\right)+{\left[\frac{R_{o}\left(n\right)}{R_{i}\left(n\right)}\right]}^{2}\left[\lambda^{D}\left(n\right)-1\right]exp\left(-nh/\tau_{f}\right)\frac{h}{\tau_{R}}R_{o}\left(t_{0}\right)-\frac{b\left(n\right)}{R_{i}^{2}\left(n\right)}h.$$

4. Update values for *c*(*n*) and *d*(*n*) using their definitions by substituting the newly derived *R_i _*and *R_o_*.

(23) $$c\left(n+1\right)=\left\{1-\frac{P\left(n+1\right)}{E\left(n+1\right)}\frac{1}{R_{i}^{o}{\left(n+1\right)}^{3}-1}\left[\left(1+\nu \right)\frac{R_{i}^{o}{\left(n+1\right)}^{2}+R_{i}^{o}\left(n+1\right)}{2}\left(1-2\nu \right)\right]\right\}\left[R_{o}\left(n+1\right)-R_{i}\left(n+1\right)\right],$$

(24) $$d\left(n+1\right)=R_{o}^{3}\left(n+1\right)-R_{i}^{3}\left(n+1\right).$$

5. Compute new values for *r_i_*, *r_o _*and λ

(25) $$r_{i}\left(n+1\right)=\frac{-c\left(n+1\right)+\sqrt{\left[4d\left(n+1\right)/c\left(n+1\right)-c^{2}\left(n+1\right)\right]/3}}{2},$$

(26) $$r_{o}\left(n+1\right)=\frac{c\left(n+1\right)+\sqrt{\left[4d\left(n+1\right)/c\left(n+1\right)-c^{2}\left(n+1\right)\right]/3}}{2},$$

(27) $$\lambda \left(n+1\right)=\frac{r_{o}\left(n+1\right)}{R_{o}\left(n+1\right)}.$$

The numerical calculations were performed using in-house Matlab codes. In all simulations, parameter *τ_R _*= 0.22 month, *D *= 2.5 and *τ_f _*= 5.6 month, initial values used in our model were listed in Table-1. The initial radii were determined by minimizing the error from experimental measurements considering both end diastolic dimension/diameter (EDD) and wall thickness.

## Results

### Changes of EDD, wall thickness and stress

**Figure 4:**
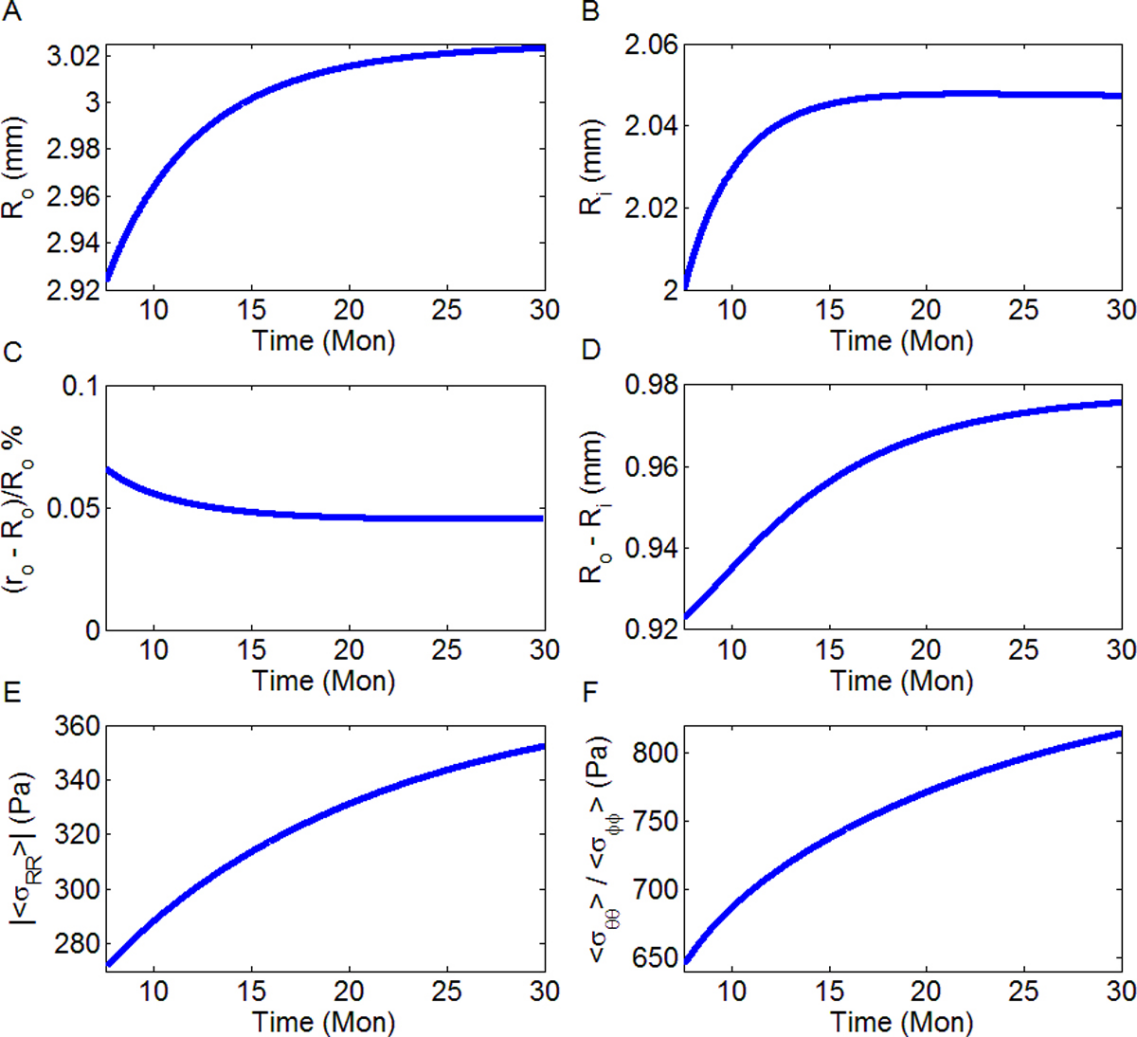
**Temporal curves of LV dimensions and stresses derived by solving the simultaneous equations**. The parameters in Eq. (14) adopted are $\tau_{R}=0.22$ mons, D=2.5 and $\tau_{f}=5.6$ mons. (A) Temporal profile of the strainless radius on the external surface of LV; (B) The internal radius change with age; (C) Percentage change between deformed and free states. They are almost identical; (D) Wall thickness that has a 5.72% thickening from young to old mice; (E) Absolute value of negative spatial mean strain in radial direction; (F) Circumferential (or meridional) stress averaged across the wall.

The numerical simulation on LV dimensional changes including EDD and wall thickness were shown in Figures 4. The interior radius increased from 2.00 mm to about 2.05 mm (see Figure 4B), which is consistent with the 2.60% increase in our mice experiments for EDD from young to senescence. None of the percentage difference between strainless and deformed radii exceeds 0.1% for all time of concern, indicating a proper adoption of small deformation Hook's law (see Figure 4C). The wall thickness started from 0.92 mm, ended with 0.98 mm and had an increase of 0.053 mm, or a 5.72% ascent (Figure 4D), which is of the same magnitude as the 13.20% increase in our experiments.

The temporal profiles of the mean *σ_RR _*and mean *σ_θθ _*(or *σ_φφ_*) across the wall were shown in Figures 4E and 4F calculating by

(28) $$\begin{array}{llll} <\sigma_{RR}> & =\frac{1}{R_{o}-R_{i}}\int_{R_{i}}^{R_{o}}\frac{PR_{i}^{3}}{R_{o}^{3}-R_{i}^{3}}\left(1-\frac{R_{o}^{3}}{R^{3}}\right)dR\; & & \;\\ & =-\frac{R_{i}\left(R_{o}+2R_{i}\right)}{2\left(R_{o}^{2}+R_{o}R_{i}+R_{i}^{2}\right)}P\; & & \;\\ & \; & \end{array}$$

(29) $$\begin{array}{llll} <\sigma_{\theta \theta }>=<\sigma_{\phi \phi }> & =\frac{1}{R_{o}-R_{i}}\overset{R_{o}}{\underset{R_{i}}{\int }}\frac{PR_{i}^{3}}{R_{o}^{3}-R_{i}^{3}}\left(1+\frac{R_{o}^{3}}{2R^{3}}\right)dR\; & & \;\\ & =\frac{R_{i}\left(R_{o}^{2}+R_{o}R_{i}+4R_{i}^{2}\right)}{4\left(R_{o}^{3}-R_{i}^{3}\right)}P\; & & \;\\ & \; & \end{array}$$

As is seen in Eq. (2), $\sigma_{RR}\leq 0$ and its contribution is a force toward inner direction. The magnitude of all these averaged stresses went up with time, which was a natural result of enhanced pressure *P*(*t*) (Figure 3A). We also showed that the magnitude of the circumferential or meridional stress was higher than that the radial (comparing Figures 4E and 4F).

### Pressure-volume relationship with aging

**Figure 5:**
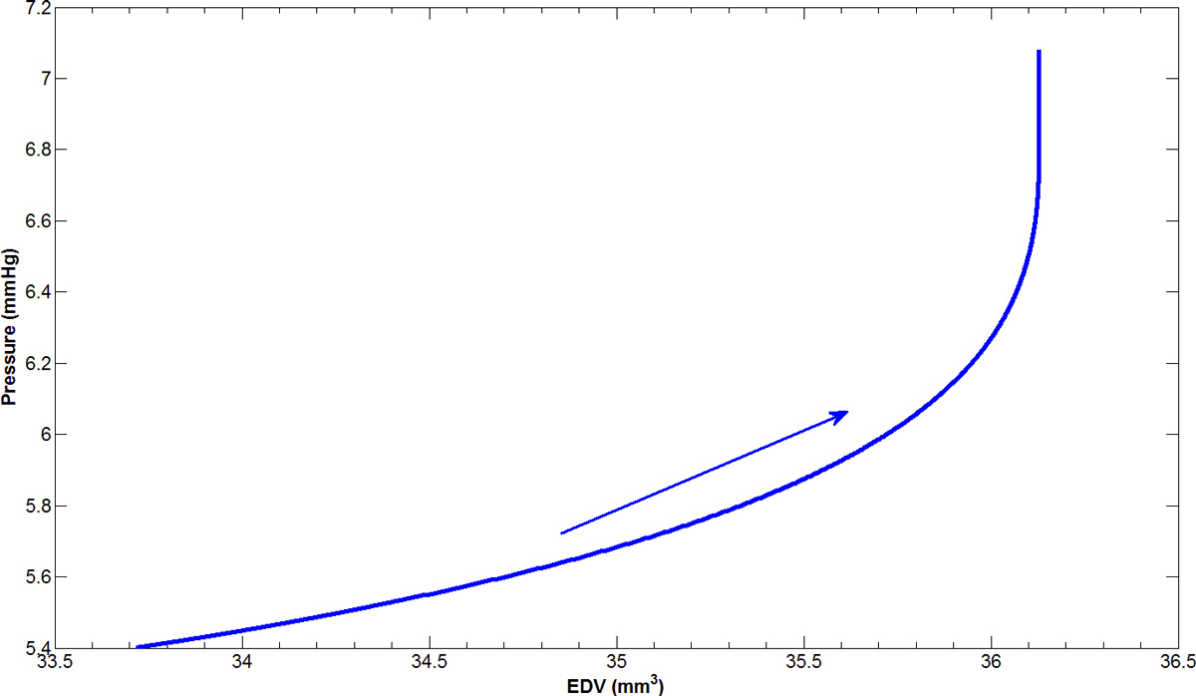
**End diastolic pressure-volume relationship with age**. The arrow implies aging direction. Both pressure and end diastolic volume go up with age.

End diastolic portion of pressure-volume loop in a cardiac cycle provides important information of passive filling for LV and therefore describes passive properties of myocardium. For example, the slope of end diastolic pressure-volume relationship (EDPVR) gives the measure of LV stiffness. We plotted end diastolic pressure-volume curve with age for C57/BL6J mice (see Figure 5), in which $EDV=\left(4/3\right)\pi r_{i}^{3}$ is the volume of the cavity. The arrow indicates the aging direction. The ratio of changes between pressure and end diastolic volume goes up with age, together with increased pressure and volume with age. It is worth noting that the end diastolic P-V relation shown in Figure 5 is a temporal progression with age, which is different from the typical one cardiac cycle P-V loop.

### Analysis of constraints of remodeling equation

We also analyzed the candidates of the tissue growth function f(t) based on assumption 2). From Eq. (12), we had: $R_{i}^{2}\left(t\right){Ṙ}_{i}\left(t\right)=R_{o}^{2}\left(t\right){Ṙ}_{o}\left(t\right)-b\left(t\right)$. So ${Ṙ}_{i}\left(t\right)\geq 0$ is equivalent as ${Ṙ}_{o}\left(t\right)\geq b\left(t\right)R_{o}^{-2}\left(t\right)$. Apply dynamic equation, i.e. equation (14), ${Ṙ}_{o}\left(t\right)=R_{o}\left(t_{0}\right)\dot{\alpha }\left(t\right)$, the inequality became: $f\left(t\right)\geq \frac{b\left(t\right)}{R_{o}\left(t_{0}\right)R_{o}^{2}\left(t\right)}$. On the other hand, wall thickness growth also gave ${Ṙ}_{o}\left(t\right)\geq {Ṙ}_{i}\left(t\right)$. Since ${Ṙ}_{i}\left(t\right)=\frac{R_{o}^{2}\left(t\right){Ṙ}_{o}\left(t\right)-b\left(t\right)}{R_{i}^{2}\left(t\right)}$, ${Ṙ}_{o}\left(t\right)\geq \frac{R_{o}^{2}\left(t\right){Ṙ}_{o}\left(t\right)-b\left(t\right)}{R_{i}^{2}\left(t\right)}$, i.e. ${Ṙ}_{o}\left(t\right)\leq \frac{b\left(t\right)}{R_{\text{o}}^{2}-R_{i}^{2}}$, substituting *R_o _*with *α *and using equation for $\dot{\alpha }$, the inequality became $f\left(t\right)\leq \frac{b\left(t\right)}{R_{o}\left(t_{0}\right)\left[R_{\text{o}}^{2}\left(t\right)-R_{i}^{2}\left(t\right)\right]}$, which led to

(30) $$\frac{b\left(t\right)}{R_{o}\left(t_{0}\right)R_{o}^{2}\left(t\right)}\leq f\left(t\right)\leq \frac{b\left(t\right)}{R_{o}\left(t_{0}\right)\left[R_{o}^{2}\left(t\right)-R_{i}^{2}\left(t\right)\right]},$$

where *b*(*t*) was a universal input function in our system and did not depend on the selection of parameters. *R_o_*(*t*_0_), i.e. initial value of *R_o_*, was a constant. However, $R_{o}^{2}\left(t\right)$ and $R_{o}^{2}\left(t\right)-R_{i}^{2}\left(t\right)$ could only be determined after the simultaneous equations being solved. Therefore, these two terms were subject to the parameters adopted in the mathematical model. However, we could find universal upper and lower bounds for the growth rate function, *f*(*t*). As we know, *R_o_*(*t*) increases with aging. Hence, $\frac{b\left(t\right)}{R_{o}^{3}\left(t_{0}\right)}$ is a natural high upper bound of *f*(*t*). By defining $S\left(t\right)=R_{o}^{2}\left(t\right)-R_{i}^{2}\left(t\right)$, it is easy to see *S*(*t*) went up with time because both inner, outer radii and wall thickness enlarge. So *S*(*t*) was maximized at 30 months which was the terminal simulation time span. To achieve the maximum surface area difference for two concentric spheres kept at a constant volume, the smaller the inner/outer radius, the bigger the value *S *is. Since both interior and exterior radii increase, the lowest possible radius at the internal surface was *R_i_*(*t *= 30 *mons*) = *R_i_*(*t*_0_). Use the mass of each component at the final time and corresponding density, the final volume was determined and Parameter *R_o_*(*t *= 30 *mon*) was calculated, which is the exterior radius in month 30 assuming the interior radius is the same as that in month 7.5, i.e. its initial value. Hence, $\frac{b\left(t\right)}{R_{o}\left(t_{0}\right)\left[R_{o}^{2}\left(t=30ṁons\right)-R_{i}^{2}\left(t=30\;mons\right)\right]}$ offers a lower upper bound. Therefore, if the following inequalities are satisfied, the selection of the radius growth rate function *f*(*t*) should give us proper remodeling trend,

(31) $$\frac{b\left(t\right)}{R_{o}^{3}\left(t_{0}\right)}\leq f\left(t\right)\leq \frac{b\left(t\right)}{R_{o}\left(t_{0}\right)\left[R_{o}^{2}\left(t=30mons\right)-R_{i}^{2}\left(t=30mons\right)\right]}.$$

And the above inequalities do not rely on a concrete system. Both sides are known functions without parameter dependence. Therefore, it is a universal condition for the selection of the type of rate function. *f*(*t*) Though this is a stringent condition, functions fall in the proposed region will guarantee the stability of the system.

**Figure 6:**
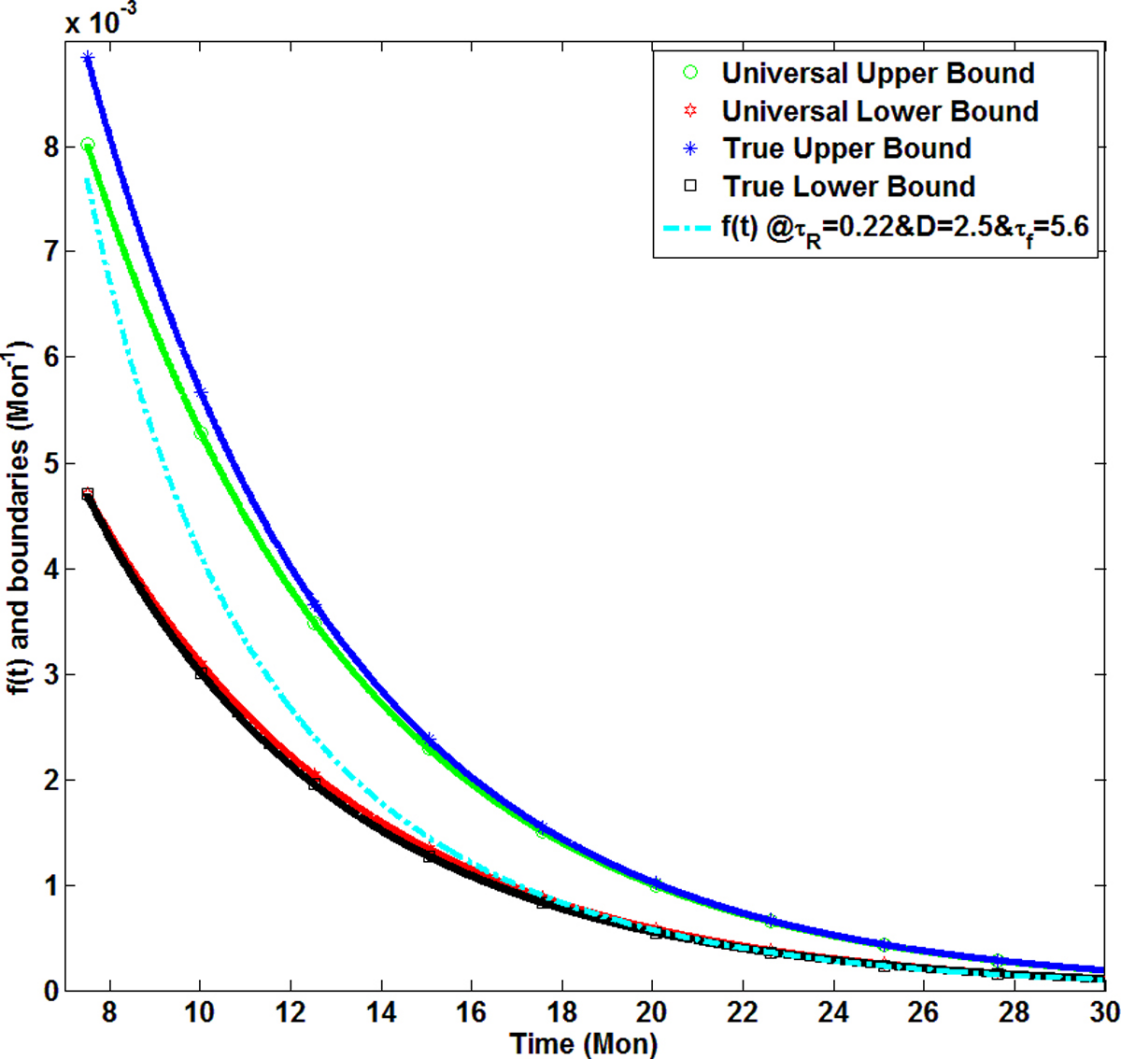
**Boundaries for the LV growth rate function **$f\left(t\right)$**to generate growing trends for internal radius and wall thickness**. The dotted cyan line in the middle is our $f\left(t\right)$ for $\tau_{R}=0.22$ mons, D=2.5 and $\tau_{f}=5.6$ mons. The star-marked blue line and square-marked black line form boundaries for our selection of parameters. Between the green curve with circle markers and red one with hexagram markers is the region satisfying conditions determined by equations (30-31).

As is shown in Figure 6, the green line with circle markers and red line with hexagram markers are the universal boundaries determined by equation (31). The blue curve with star markers and the black one with square markers are true upper and lower bounds respectively for *f*(*t*) calculated in equations (30) for the set of parameters listed above.

In our simulations, parameters *τ_R _*= 0.22 months, *D *= 2.5 and *τ_f _*= 5.6 months were chosen by minimizing the deviations of computed EDD and wall thickness from measurements at senescent age, i.e. (*R_i _*- *R_i_*,*_exp_*)^2 ^+ (*WT *- *WT_exp_*)^2^, in which WT means wall thickness. The growth function *f*(*t*) determined by these parameters, the dotted cyan curve, falls in the bounded region. The analysis of the boundary of the growth function provided a standard procedure for us to determine the possible sets for parameters with or without simulation results, which should be able to be applied in the modeling of age-dependent remodeling process of different species given experimental data, e.g. rat, dog or even human. In addition, if a growth function *f*(*t*) of this form is given, we can also use this boundary condition to predict whether LV geometry and wall thickness will monotonically increase or not.

## Discussion

In this study, we established the first analytical mathematical model to quantify long-term LV geometric remodeling dynamics with aging by applying thick-wall theory and stretch-induced tissue growth postulate. This model addressed the temporal progression of cardiac aging and is an advancement from most current models that analyze the static equilibrium of LV remodeling and our previous thin-wall model of LV remodeling. In addition, we analytically determined the boundary for the tissue growth rate to guarantee the stability of the remodeling. This boundary might provide a reference for experimental measurement to examine the remodeling outcomes. Further, the parameters in the mathematical model were determined by using our experimental results from over 140 C57/BL6 mice. Some parameters and initial conditions were selected by minimizing the error between our computational predictions and experimental measurements. In addition, the model was validated by comparing the predictions of internal and external radii and wall thickness in diastole to experimental results. The predicted LV geometry trends were consistent with the experimental results. Thus, this study is a real integration of computational and experimental approaches for model establishment and validation using mice data. This approach and the proposed model can be applied to establish a cardiac aging model for human in the future.

The P-V curve in Figure 5 shows that both pressure and end diastolic volume escalate with age. This has been observed in a LV P-V relationship experiment on rat aging study by Pacher and colleagues [25]. In their study, LV end diastolic volume, LV end diastolic pressure, and the slope of EDPVR were greater in senescent than in young rats. In another experimental aging study on C57BL/6 female mice [26], the pressure was augmented in 16-month-old mice compared to 6-month-old group. In our experimental study, we observed increased LV volume with age. These experimental results confirmed our computational predictions.

Though our computational results follow the trend of LV geometry changes, the proposed model has some limitations. First, we assumed the LV to be a spherical shell. With this assumption, all directions grow at the same pace to maintain the spherical symmetry. Sphere is a special geometry that requires minimum linear dimension for a constant volume. However, LV does not have a spherical geometry in reality. In addition, we assumed that LV was composed of elastic, isotropic, and homogeneous materials and applied the linear mixture theory to calculate the Young's modulus of the myocardium. Though the assumptions simplify the mathematical model and the computational analysis, such simplification might lead to prediction errors. From our computational results, the radii growth calculated are 3.43% for external, 2.37% for internal radius, and 5.72% for wall thickness collected from C57/BL6 mice. Some of these values were small compared to our echo experiment on C57/BL6 mice, in which EDD grows 2.60% and wall thickness is up 13.20% from young to senescent. In addition, our previous experimental work on CB6F1 mice reported EDD and wall thickness have 12.73 and 13.00% growth, respectively, in the LV between the groups of young and senescent [27]. This discrepancy may be originated from the simplification of the geometry we chose or the prediction of the myocardium stiffness. Future experiments will build on these limitations to improve our results by incorporating more realistic geometry and taking more advanced techniques to determine LV stiffness.

## Conclusions

We have established the first mathematical model to study age-related temporal-spatial LV remodeling by adopting thick-wall theory and stretch-induced tissue growth theory. IInputs of the mathematical model were real experimental data including temporal profiles of LV mass, collagen content change, and pressure across LV, which were obtained by over 140 mice. The established model captured the major property of LV remodeling with age and yielded predicted results of LV geometry progressions in mice comparable to experimental results.

## Competing interests

The authors declare that they have no competing interests.

## Authors' contributions

Y.F.J and M.L.L designed the research; Y.A.C, A.V, H.C.H, and M.L.L performed the experiment, Y.F.J, T.Y, and Y.W performed the computational analysis and simulation. T.Y, H.C.H, M.L.L, and Y.F.J analyzed the results and wrote the manuscript.
